# Ulcerative colitis: Recent advances in the understanding of disease pathogenesis

**DOI:** 10.12688/f1000research.20805.1

**Published:** 2020-04-24

**Authors:** Ross J Porter, Rahul Kalla, Gwo-Tzer Ho

**Affiliations:** 1 Edinburgh IBD Science Unit, Centre for Inflammation Research, Queens Medical Research Unit, University of Edinburgh, 47 Little France Crescent, Edinburgh, EH16 4TJ, UK

**Keywords:** Ulcerative colitis, Inflammatory Bowel Disease, Inflammation, Mucosal Immunology, Pathogenesis

## Abstract

Inflammatory bowel diseases are common, complex, immune-mediated conditions with a sharply rising global prevalence. While major advances since 2000 have provided strong mechanistic clues implicating a de-regulation in the normal interaction among host genetics, immunity, microbiome, and the environment, more recent progress has generated entirely new hypotheses and also further refined older disease concepts. In this review, we focus specifically on these novel developments in the pathogenesis of ulcerative colitis.

## Introduction

The Inflammatory Bowel Diseases (IBDs), namely Ulcerative Colitis (UC) and Crohn’s disease (CD) ( Table 1), are chronic immune-mediated conditions with a high prevalence in developed countries (>0.3%) and rapidly increasing incidence in newly industrialised countries (annual percentage change +14.9%) ^1, 2^. Global prevalence is projected to affect up to 30 million individuals by 2025 ^3^. Since its original description by Samuel Wilks in *Morbid appearances in the intestine of Miss Bankes* in 1859, the notably consistent features of UC that at once appear to be such strong clues have not yet led to a clear understanding of disease pathogenesis ^4^. These clinical features include the almost-universal involvement of the rectum (the lowest part of the colon) as the first site where inflammation starts and the distinctively confluent nature of inflammation that ends with an abrupt demarcation and transition into normal colonic mucosa. Smoking is protective, and UC often presents after smoking cessation ^5^. Furthermore, the development of appendicitis is protective against UC. On the other hand, UC (like CD) is clinically heterogeneous: only 30% and 15% of patients have extensive (affecting more than half of the colon) or aggressive (patients rapidly become unwell with features of systemic upset) colitis, respectively ^6^. Approximately half of patients may develop a more complicated disease course, some by virtue of not responding to drug treatments ^7– 9^. Hence, like many complex diseases, diverse aetiological factors shape the initiation of UC and impact subsequent disease course and severity ( Table 2).

**Table 1.** Summary of clinical features of Crohn’s disease and ulcerative colitis.

	Crohn’s disease (CD)	Ulcerative colitis (UC)
Incidence of inflammatory bowel disease (IBD)		
Sex	Higher incidence in females than in males	Equal incidence in males and females
Global prevalence	High incidence of CD in developed countries with high prevalence	UC emerged before CD in developed countries; UC is more prevalent in newly industrialised countries
Clinical presentation		
Symptomology	Chronic diarrhoea, abdominal pain, fever, malnourishment, fatigue, and weight loss	Most commonly bloody diarrhoea with abdominal pain, urgency, and tenesmus; haematochezia is more common in UC
Serological markers	Antibodies to microbiota including anti- *Saccharomyces cerevisiae* antibodies; also, anti-OmpC, anti-I2, and anti-Cbir1 antibodies and antibodies against exocrine pancreas	Anti-neutrophil cytoplasmic antibodies; also, antibodies to goblet cells
Gross pathology and histopathology		
Affected areas	Can affect the entire gastrointestinal tract (from mouth to anus); terminal ileum is often implicated	Affects the colon with potential backwash ileitis or rectal sparing in longstanding disease
Pattern of inflammation	Often patchy and discontinuous cobblestone pattern of inflammation with skip lesions	Continuous inflammation extending from the rectum proximally, often with a separate caecal patch
Penetrance	Transmural inflammation of the entire bowel wall	Inflammation restricted to the mucosal and submucosal layers (except in fulminant colitis)
Histopathology	Thickened colon wall with non-caseating granulomas and deep fissures Fibrosis, lymphangiectasia, mural nerve hypertrophy, and Paneth cell metaplasia can sometimes be observed Granulomas are present in about half of Crohn’s patients	Distorted crypt architecture with shallow erosions and ulcers Goblet cell depletion, pseudopolyps, submucosal fibrosis, and mucosal atrophy can sometimes be observed
Complications		
IBD complications	Fistulas, strictures, perianal abscesses, and colonic and small bowel obstruction (from strictures, adhesions, or carcinoma)	Fulminant colitis, toxic megacolon perforation, and haemorrhage Colorectal cancer is more common in UC

**Table 2.** Overview of recent advances in ulcerative colitis (UC).

The current platform of UC pathogenesis
**Genetics** • Most genetic factors (67% of susceptibility loci) are shared between UC and Crohn’s disease (CD) • Sixteen human leukocyte antigen (HLA) allelic associations (mostly class II) are described for UC • Outwith the HLA region, the *ADCY7* gene has the strongest association with UC • UC-specific genes implicate epithelial dysfunction • There is low disease hereditability in UC (6.3% in monozygotic twins)
**Environment** • UC incidence rises before CD and this is associated with Westernisation • Westernisation factors—urban lifestyle, pollution, diet, antibiotics, better hygiene, and fewer infections—are associated with UC • Appendicitis and smoking are protective in UC; smoking cessation can precede UC • Patients with UC have a 30% increased risk of developing Parkinson’s disease
**Microbiota** • The UC gut microbiome, virome, and mycobiome is less diverse over time • Faecal microbial transplantation is effective in UC • It is not known if dysbiosis is a consequence, or initiator, of inflammation • There is depletion of protective (Ruminococcaceae and Lachnospiraceae) and enrichment of inflammatory (Enterobacteriaceae and Fusobacteriaceae) microbes
**Epithelial barrier** • An impaired epithelial barrier is a pathogenic factor for UC • An innate “at risk” barrier-specific genetic phenotype where exposure to additional injurious stimuli, such as non-steroidal anti-inflammatories and dietary components such as emulsifiers, may be the second trigger that precipitates colitis
**Immune response** • Neutrophils are “first responder” cells and undergo inflammatory cell death, which drives inflammation • Innate immune responses (neutrophils/macrophages) may promote a pathogenic adaptive (likely T-cell driven) response • How HLA allelic associations influence antigen presentation is not fully understood • UC immunity is more complex than simply a non-classical Th2 response given newly discovered Th19 and Th17 responses and effective interleukin (IL)-23 blockade therapy
New progress in the pathogenesis of UC
**Mitochondria** • Mitochondriopathy is a pathogenic process in UC • Loss of mitochondrial homeostasis leads to defective energy production, increased oxidative stress, and the release of pro-inflammatory damage-associated molecular patterns
**Single-cell data** • New colonic epithelial cell subsets have been identified that can sense colonic luminal pH and set the epithelial cGMP tone in response; goblet cell remodelling also has important implications • Strong compartmentalisation around inflammatory monocytes and novel network hubs around the poorly characterised *CD8 ^+^IL17 ^+^* T cells and microfold-like (M) cells are observed in UC • In some patients, inflammation-associated fibroblasts (IAFs) are expanded, enriched with many genes associated with colitis, fibrosis, and cancer • One of the most enriched genes in IAFs is oncostatin M ( *OSM*); high mucosal *OSM* is associated with poor response to anti-tumour necrosis factor

## The current platform of UC pathogenesis

**Figure 1.**
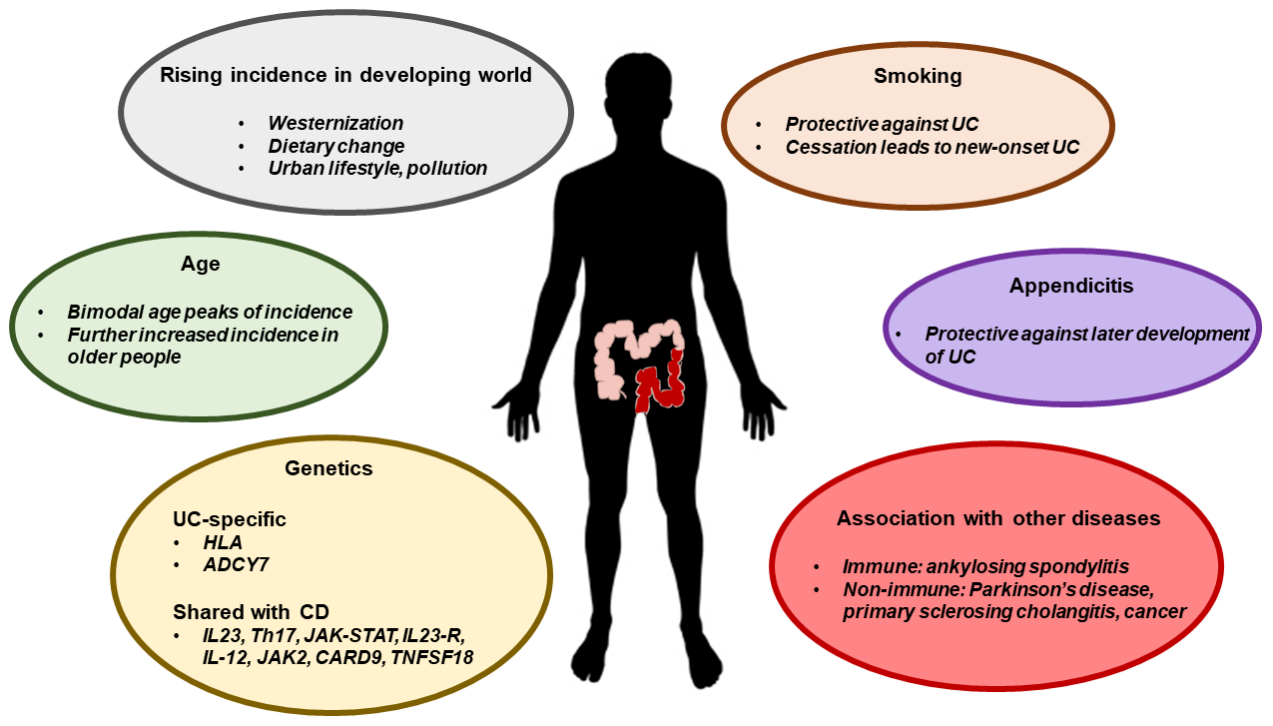
General factors associated with increased susceptibility of UC. CD, Crohn’s disease; UC, ulcerative colitis.

**Figure 2.**
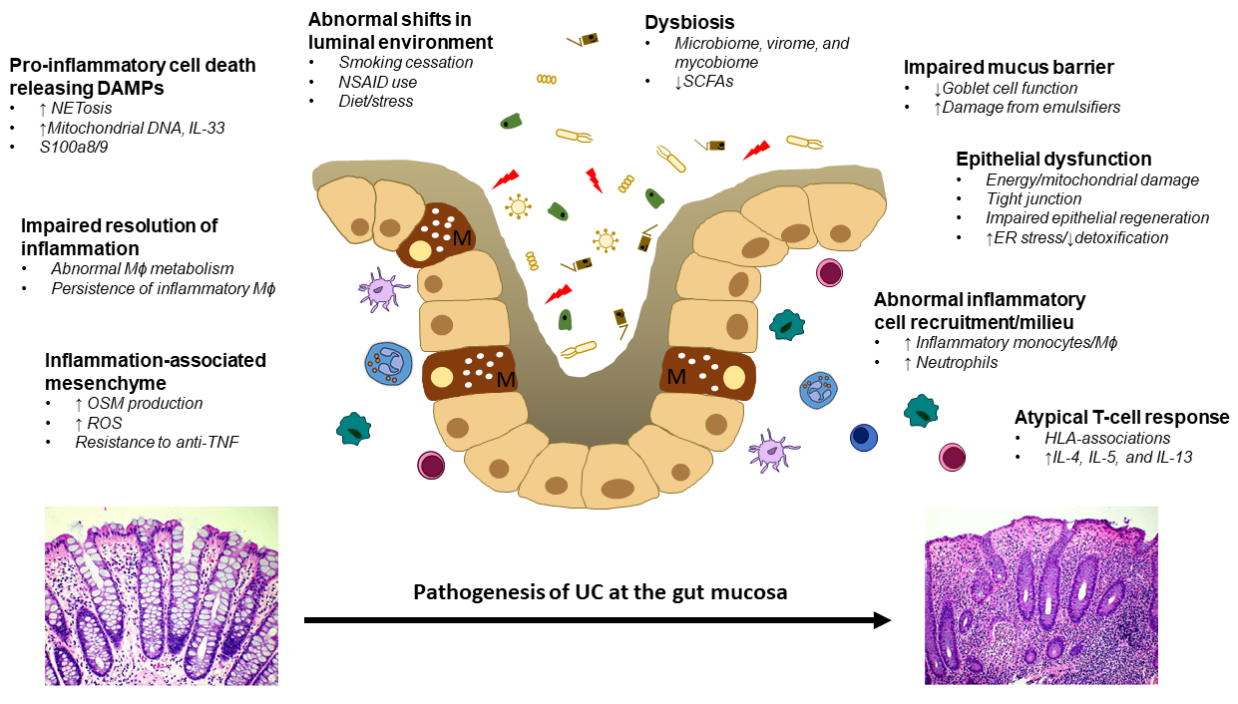
Molecular mechanisms involved in the development of mucosal inflammation in UC. DAMPs, damage-associated molecular patterns; ER, endoplasmic reticulum; HLA, human leukocyte antigen; IL, interleukin; Mɸ, macrophage; NSAID, non-steroidal anti-inflammatory drug; OSM, oncostatin M; ROS, reactive oxygen species; SCFA, short-chain fatty acid; TNF, tumour necrosis factor; UC, ulcerative colitis.

A widely accepted framework suggests a complex contribution of environmental and host factors that increase the susceptibility of developing UC, and disease onset is triggered by events that perturb the mucosal barrier, alter the healthy balance of the gut microbiota, and abnormally stimulate gut immune responses. Here, we discuss the general aetiological factors that increase the risk of developing UC ( Figure 1) and review the molecular underpinnings of the abnormal inflammatory process in this disease ( Figure 2). We briefly cover the genetic, environmental, immune, and microbiome factors that currently frame our understanding of UC pathogenesis.

### Genetics

Genetic studies (including genome-wide association [GWA], whole genome sequencing [WGS], and fine mapping studies) have been particularly successful in identifying 260 susceptibility loci (both common and rare genetic variants) associated with IBD ^10– 14^. There are several key findings. Firstly, most genetic factors are shared between UC and CD. In an initial analysis of 15 GWA datasets, Jostins *et al.* showed that 110 out of 163 (67%) susceptibility loci were associated with both UC and CD ^11^. These shared genes encode both innate and adaptive immune pathways, cytokine signalling, and immune sensing (e.g. *IL23-R, IL-12, JAK2, CARD9, TNFSF18,* and *IL-10*). Many of these genes (70%) are also shared with other autoimmune diseases such as ankylosing spondylitis and psoriasis. Secondly, the strongest genetic signals within UC-specific loci are associated with the human leukocyte antigen (HLA) region in chromosome 6. Sixteen HLA allelic associations (mostly class II) are described for UC, including HLA DRB1*01*03 for IBD colonic involvement on deeper fine mapping genetic analysis ^15^. Further analyses show that these are associated with colonic involvement for UC and CD ^16^. It is of interest to note that HLA allelic associations with extensive and aggressive UC have been noted even prior to GWA studies ^17^. Recent WGS of nearly 2,000 UC patients identified a new but rare missense variant (present in 0.6% of cases) in the adenylate cyclase 7 gene ( *ADCY7*) that doubles the risk of UC ^12^. Outwith the HLA region, the *ADCY7* gene has the strongest genetic association observed with UC. ADCY7 is one of a family of 10 enzymes that convert ATP to the ubiquitous second messenger cAMP. In addition to this, many UC-specific genes are involved in the regulation of epithelial barrier function (further discussed below). Thirdly, despite the identification of many susceptibility loci, genetics explain only 19% of disease heritability in UC ^18^. The concordance rate amongst monozygotic twins for UC is only 6.3% (compared to nearly 60% in CD). Collectively, genetic factors confer a small but definite increase in susceptibility for UC. Many individuals, however, have no genetic predisposition when assessed by a polygenic risk score that accounts for all of the susceptibility loci ^19^. This suggests a key role for aberrant adaptive immune responses and epithelial barrier dysfunction in UC disease pathogenesis. Non-genetic factors (notably epigenetics ^20, 21^) may also play an important role.

### Environmental factors

The rapid rise of UC incidence in newly industrialised countries suggests that environmental factors are important ^1^. This parallels the patterns observed in the Western world during the early 20 ^th^ century. Specifically, UC appears first in urban areas, its incidence rising rapidly then slowing; subsequently, CD incidence rises and eventually approaches that of UC ^22^. Westernisation is accompanied by new urban lifestyle, exposure to pollution, change in diet, access to antibiotics, better hygiene, and fewer infections, all considered as general contributory factors ^23^. Notwithstanding this, more specific environmental factors associated with UC have been known for some time. The strongest example is seen in the protective effect of cigarette smoking and the notable observation of new-onset UC in individuals who stop smoking. The global patterns of smoking and IBD are changing; an increasingly large former smoker population with UC in China is suggestive of a rapid expansion of the at-risk population ^24^. The anti-inflammatory effect conferred by cigarette smoking in UC is intriguing and may be mediated by carbon monoxide ^25^. Further examples include the protective effect of appendicitis against future development of UC ^26, 27^, the bimodal incidence with a second peak associated with older age in men ^28^, and, more recently, the curious association with Parkinson’s disease (another condition associated with non-smoking and old age) ^29, 30^. These all provide more specific aetiological insights into the development of UC. Epidemiologic data have shown a potential protective effect of high dietary n-3 polyunsaturated fatty acids (PUFAs), present in oily fish ^31^, and a diet high in red meat in the development of UC ^32– 34^.

### Gut microbiota

The IBD gut microbiome is significantly less diverse and stable over time, as recently extensively characterised in the Integrative Human Microbiome Project ([iHMP], where 132 IBD and healthy individuals were followed up longitudinally for 1 year) ^35^ and demonstrated in a case-control study involving 1,800 IBD and irritable bowel syndrome patients ^36^. A depletion of protective bacteria such as short-chain fatty acid (SCFA)-producing Ruminococcaceae and Lachnospiraceae that coincides with an expansion of pro-inflammatory microbes such as Enterobacteriaceae, including *Escherichia coli*, and Fusobacteriaceae has been noted ^37, 38^. These changes, however, are less obvious in UC compared to CD ^39^. It is not known if dysbiosis is a consequence of, or plays a causal role in, gut inflammation in UC. In this regard, the virome and mycobiome are also less diverse in UC ^40– 43^. In the longitudinal iHMP, microbiome patterns did not predict the likelihood of a disease flare. To add to the complexity, a further study in UC showed that microbial abundance did not necessarily correlate with transcriptional activity ^44^. Therapeutically, however, faecal microbial transplantation (FMT) from healthy donors can treat UC. There are four controlled positive FMT clinical studies ^45– 49^. The restoration of microbial diversity, including bacterial species responsible for SCFA production in donor stool, has been suggested as an important contributor ^46, 50^. Hence, one of the main effects of dysbiosis in UC is likely to be a reduction in epithelial health or a state of epithelial dysfunction that further primes innate susceptibility to UC. In support of this, faecal diversion away from the rectum worsens inflammation, giving rise to “diversion colitis” in UC; the opposite is true for CD, where faecal diversion improves inflammation ^51^.

### Epithelial dysfunction

With the histologic observation of subepithelial inflammation, many studies implicate an impaired epithelial barrier as a pathogenic factor for UC. This is through either altered or impaired secretion (e.g. of antimicrobial peptides, damage-associated molecular patterns, or mucus) or physical defects (e.g. from disruption of epithelial tight junctions or defective regeneration or detoxification) ( Text box 1) ^52, 53^. GWA studies show UC-specific susceptibility genes that regulate epithelial morphogenesis ( *hepatocyte nuclear factor 4 α, Hnf4α* ^54^), adherens junction stability via E-cadherin ( *CDH-1)*, basement membrane anchoring and stability (via laminins, *LAMB-1*, and extracellular matrix, *ECM1*), tight junction assembly (guanine nucleotide binding protein alpha 12, *GNA12*), ion transport *(*solute carrier family-26 *, SLC26A3)* ^55^, and epithelial health via endoplasmic reticulum stress (orsomucoid-1-like gene 3, *ORMDL3)* ^56^. Of interest, a protein truncating genetic variant in *RNF186*, a single-exon ring finger E3 ligase with strong colonic epithelial expression, protects against UC; however, the underlying mechanism is not yet clear ^14, 57^. Hence, there is a potentially innate “at risk” phenotype where exposure to additional injurious stimuli such as non-steroidal anti-inflammatories ^58^ (that reduce the synthesis of protective prostaglandins) and dietary components such as emulsifiers (that reduce the thickness of the mucus layer) ^59^ may be the second trigger that precipitates colitis. As discussed earlier, dysbiosis results in loss of SCFA production ^35^, which is essential for epithelial energy provision, mucus production, and proliferation in the colon. Hence, clinical trials involving butyrate ^60^, propionic acid ^61^, prebiotics ^62– 66^, and L-carnitine ^61^, which facilitate SCFA transport, have demonstrated some efficacy in treating UC ^67^. During active UC, key pro-inflammatory cytokines such as tumour necrosis factor-alpha (TNF-α), interferon (IFN)-γ, and interleukin (IL)-13 have direct deleterious effects on epithelial barrier integrity ^68, 69^. Drugs that maintain remission in UC, such as mesalazine, may exert some of their therapeutic effect by maintaining epithelial health ^67^. Hence, protecting the “at risk” or restoring colonic epithelial health may be a viable strategy to maintain long-term remission in UC.

**Text box 1. Mucosal compartments of the gut wall**

**Secreted mucus barrier**

Mucus plays dual roles as a lubricant and a physical barrier between luminal contents and the intestinal epithelium. In the colon, an inner layer provides a bacteria-free environment adjacent to the epithelium, and the luminal less-viscous layer harbours the gut microflora.

**Colonic epithelium**

The single layer consists of intestinal epithelial cells (IECs), mostly absorptive colonocytes connected by tight junctions, interspersed with specialised epithelial lineages, including secretory goblet and enteroendocrine cells (EECs).

**Lamina propria**

The mucosal compartment beneath the epithelium supported by loose connective tissue and populated by resident immune cells such as macrophage and dendritic cells, along with mesenchymal cells.

**Mesenchymal (stromal) cells**

Mesenchymal cells of the intestinal lamina propria are a heterogeneous population of non-hematopoietic, non-epithelial cell types that contribute to the regulation of innate immunity and epithelial barrier maintenance with major intestinal tissue stromal cell subsets such as fibroblasts, α smooth muscle actin (α-SMA)-expressing myofibroblasts, and perivascular pericytes.

### Abnormal immune response: innate

In active UC, there is a complex inflammatory milieu of innate and adaptive immune cells infiltrating the lamina propria. Neutrophils, the short-lived “first responder” cells, are recruited in abundance with characteristic histology of “crypt abscesses” in UC, where neutrophils transmigrate across the colonic epithelium and die within the colonic crypts ^70^. The UC inflammatory environment promotes neutrophil survival (potentially via HIF-1 and hypoxia) ^71, 72^. This increased survival escalates its inflammatory action and tissue damage (via many means, including the release of serine and matrix metalloproteases, reactive oxygen species, and pro-inflammatory cytokines) ^73^. The high number of neutrophils undergo uncontrolled pro-inflammatory cell death (necrosis, necroptosis, and NETosis), which potentiates and amplifies the pro-inflammatory environment ^74, 75^. This is supported by high levels of s100a8/9 proteins (or calprotectin), usually found in neutrophils, that are released in blood and stool ^76– 78^ and a prominent serological response to self perinuclear anti p-neutrophil cytoplasmic antibodies (pANCA) in UC, both likely indirect indicators of uncontrolled neutrophil cell death ^79^. Neutrophil extracellular traps (NETs) can act as a sump for immunogenic molecules that sustain the inflammatory response ^75^. Hence, there is a rational paradigm that, following disease initiation, the preceding wave of innate inflammatory neutrophils and monocytes (with their pro-inflammatory cytokine repertoire, e.g. IL-1 family, IL-6, and TNF-α) creates an inflammatory milieu (nutritional, metabolic, and cytokine) that promotes a pathologic adaptive (likely T-cell) immune response ^80^. Such a milieu can also shape newly arriving inflammatory monocytes, monocyte–macrophage function, their survival, and their phenotype, and further factors that influence the host’s ability to resolve inflammation, restore homeostasis, and repair the UC mucosa ^81, 82^.

### Abnormal immune response: adaptive

UC’s strong genetic associations with HLA (mostly class II) suggest that abnormal antigen(s) driving the aberrant T-cell response, which then further shape the pathologic cytokine milieu, are likely to be a crucial causative factor. How HLA influences commensal and/or self antigen presentation (and the identities of these) to T cells and thereafter downstream pathogenic T-cell response remains unclear and challenging. Approaches to study, screen, and define T-cell epitopes have improved considerably and progress is likely ^83^. Traditionally, UC is associated with a Th2 response with high IL-4, IL-5, and IL-13, whereas CD has a more dominant Th1/Th17 response ^84^. Earlier studies that show less IL-4 in UC, with CD1d-restricted natural killer T-cells producing IL-13, point to a non-classical Th2 response ^85^. Some recent developments have overtaken this area. These include the identification of IL-23 as a key driver of Th17 responses ^86^, genetic associations with IL-23 and its related genes ^11, 87^, and the presence of Th17 ^88^ (and Th9 ^89^) cells in UC. The Th2 angle becomes less clear where anrukinzumab (a drug that blocks IL-13 by binding with IL-4Ra, a shared subunit for IL-13 and IL-4 receptors) ^90^ and tralokinumab (a drug that blocks binding to both IL-13Ra and IL-13Ra2) are not effective in UC ^91^. Blocking IL-23, however, is effective in UC, e.g. mirikizumab (anti-p19 subunit of IL-23) ^92^ and ustekinumab (anti-p40 subunit of IL-23) ^93^. The example of anti- TNF treatment first used in CD and then shown to be equally effective in UC ^94^ demonstrates that basing a translational approach on pure Th-cytokine profile may be oversimplified. Furthermore, although CD4 T cells are considered to be more important in IBD pathogenesis, it is CD8 T cell transcriptomic signatures that have been found to influence whether UC and CD adopt a more aggressive disease course (in this study, CD4 T signatures were not useful) ^95^. New data characterising the adaptive immune populations at a transcriptomic (and at a single cell) level ^96^ will yield many more new insights. The recent discovery of innate lymphoid cells (ILCs) ^97, 98^ as a further mediator of IL-23-driven inflammatory response in the colon ^99^ is a further new dimension in UC.

## New progress in the pathogenesis of UC

### The mitochondria and UC

Recent progress has been driven by a strong focus on direct studies on the inflamed mucosa specifically in newly diagnosed or drug-naïve individuals ^38, 100, 101^. Of interest, using a bulk RNAseq approach in 206 newly diagnosed paediatric UC individuals (PROTECT study), Haberman *et al.* showed a significant reduction in the expression of mitochondrial genes that encode the oxidative phosphorylation chain (responsible for energy production) and nuclear encoded genes such as *PPARGC1A* (responsible for mitochondrial biogenesis), implicating mitochondriopathy as a pathogenic process in UC ^100^. Mitochondria are intracellular double-membrane-bound organelles with many essential physiological roles such as in energy production and the regulation of cell death and immune responses ^102^. In the last 10 years, many seminal studies have highlighted the mitochondria as the major previously unknown “jigsaw piece” in inflammation ^103^. Mitochondrial dysfunction has long been implicated in UC, as far back as 1980 ^104, 105^ (reviewed by Novak *et al*. ^106^), but new data from the last 3 years have re-focused this concept ^100, 107, 108^. Such dysregulation of genes that control mitochondrial function have been shown in earlier colonic microarray studies in UC ^109^.

Functional studies show that mitochondria are sited in a uniquely damaging environment (in the colon, more so than other tissue sites) ^107, 110^. Loss of mitochondrial homeostasis (including mitophagy and the autophagic removal of damaged mitochondria—IBD GWAS susceptibility genes *PARK7* and *LRRK2*) can lead to defective energy production ^111^, increased mitochondrial oxidative stress ^107^, and even the release of mitochondrial products (mitochondrial DNA) as pro-inflammatory DAMPs ^108, 112^. These lines of evidence contribute to key UC themes such as epithelial dysfunction, the pro-inflammatory mucosal milieu, and direct triggers of the inflammatory response. Such convergence of data has culminated in new approaches in targeting the pro-inflammatory mitochondria, for example mitochondrial anti-oxidant therapy in active UC.

### Single cell profiling of the inflamed UC mucosa

Single cell RNA sequencing (scRNA) technology was developed in 2009 before becoming more widely available in 2014. It provides a comprehensive analysis and census of the cell populations (“who is all there?”) in a complex inflamed UC mucosal milieu ^113^. In UC, three recent scRNA studies (Parikh *et al*. ^114^, Smillie *et al*. ^96^, and Kinchen *et al.* ^115^—scRNA analyses on colonic epithelium, whole layer, and mesenchyme, respectively) have provided some compelling insights ^96, 114^. These studies have identified new and rare cell types, unique cell-type-specific expression, and deep cell–cell interaction and cell lineage relationships. Secondly, mucosal compartments that have previously received less attention—notably, the colonic mesenchyme—are now implicated as key mediators of inflammation ^116^. Thirdly, they show entirely new disease angles and have unexpectedly reinvigorated some older mechanistic theories. We highlight the key findings below.

***Colonic epithelium: novel cell population and cell-specific changes.*** A main question is whether there are specific subsets of colonic epithelial cells that display intrinsic molecular pathology that can be pathogenic drivers in UC. Both scRNA studies identified a previously unknown epithelial cell population characterised by distinct expression of the calcium-sensitive chloride channel bestrophin-4 ( *BEST4*), the protease cathepsin E, and the *OTOP2* gene. Intriguingly, this colonocyte likely has the ability to sense pH in the luminal environment and to set the epithelial cGMP tone in response. Smillie *et al.* showed that BEST4 ^+^ enterocytes are distinct from epithelial cells and they are also enriched in genes including otopetrins 2 and 3 ( *OTOP2/3*), proton channels that detect pH and underlie sour taste perception, and carbonic anhydrase VII ( *CA7*). In another novel finding, Parikh *et al.* demonstrated a positional remodelling of goblet cells that coincides with downregulation of *WFDC2*, an anti-protease molecule that is expressed by goblet cells and inhibits bacterial growth. *In vivo,* WFDC2 preserves the integrity of tight junctions between epithelial cells and prevents invasion by commensal bacteria and mucosal inflammation. WFCD2 has been proposed as a regulator of innate immunity through inhibition of serine and cysteine proteases ^117^.

***Colonic epithelium: intrinsic changes associated with UC inflamed and non-inflamed mucosa.*** The sharp demarcation between inflamed and non-inflamed UC mucosa in the colon provides the unique opportunity for scRNA approaches to find distinct changes that may explain this transition from normal to affected mucosa. Interestingly, both areas exhibit many similar dysregulated gene expressions. This suggests a role for mucosal epigenetics: the transcriptional signature of UC precedes inflammation, arises as the result of a dominance of regenerative over damage cues or even as a protective mechanism in anticipation of damage, and persists after resolution. All epithelial subtypes in the inflamed UC mucosa showed upregulation of several inflammatory pathways, notably IFN-γ signalling and cytokine production. Epithelial cells downregulated metabolic processes and induced genes that are needed to produce reactive oxygen species and for microbial killing. Absorptive and secretory progenitor cells upregulated differentiation and cell migration pathways, which suggests an active attempt to repair colitis-induced damage.

***Colonic immune cell population: dominant functional cellular hubs.*** In Smillie *et al*.’s study that explored the overall colonic immune cell population, cell–cell interaction analyses in the inflamed UC mucosa showed strong compartmentalisation around inflammatory monocytes and novel network hubs around the poorly characterised *CD8 ^+^IL17 ^+^* T cells and microfold-like (M) cells that are usually rarely found in the healthy colon. *CD8* ^+^ *IL17* ^+^ T cells induce *IL17A/F*, *IL23R*, and cytotoxic, co-stimulatory, and co-inhibitory programs in UC. M cells are typically associated with lymphoid tissue in the human small intestine, where they are important for recognition of the gut microbiota but are rarely found in the healthy colon ^118^. A further striking cell–cell interaction hub is centred on a mesenchymal subset of inflammation-associated fibroblasts (IAFs) ^96^. In some UC patients, IAFs are expanded nearly 190-fold and enriched with many genes associated with colitis, fibrosis, and cancer (including *IL13RA2*).

***Colonic mesenchyme: a newly identified inflammatory component contributing to an anti-tumour necrosis factor response.*** In the mesenchyme-focused scRNA study, Kinchen *et al.* identified a distinct activated mesenchymal cell population that expressed TNF superfamily member 14 ( *TNFSF14*), fibroblastic reticular cell-associated genes, IL-33, CCL19, and lysyl oxidases ^115^. One of the most enriched genes in IAFs is oncostatin M ( *OSM*), a putative risk gene, and its receptor *OSMR ^10^*. In an earlier study ^38, 119^, West and colleagues identified significant overexpression of *OSM* in inflamed IBD mucosa ^116^. OSM is part of the IL-6 cytokine family that can induce JAK-STAT, phosphatidylinositol-3-kinase (PI3K), and mitogen-activated protein kinase (MAPK) downstream signalling pathways. Further characterisation showed that *OSMR* is highly expressed in the mesenchyme (as later also shown to be the case). Using UC clinical trial datasets on anti-TNF treatment (infliximab and golimumab), high mucosal *OSM* expression is associated with poor response to anti-TNF ^120, 121^.

***Future insights from scRNA studies.*** These recent studies provide a vast “library reference” level amount of data that the IBD research community is only beginning to assimilate and understand. Tantalising new discoveries such as epithelial pH sensing, the roles of new enterocytes marked by BEST4 ^+^, and colonic anti-bacterial responses mediated by *WFDC2* and *CD8* ^+^ *IL17* ^+^ T cells will require more detailed studies. These are early days of moving from census to understanding function and biology. Other leads such as OSMR blockade and CCL9 inhibition are nearer to translation as potential therapeutic targets. The International Human Gut Atlas Project ( https://helmsleytrust.org/rfa/gut-cell-atlas) will generate an even larger compendium of scRNA data in the next 5 years. Rationalising these enormous data (with other -omics datasets, e.g. genetics and microbiome), or, in lay-terms, how we combine the knowledge of “what and where are the cells?” with “what genes?” and “what bacteria?”, will be both challenging and exciting ^122^.

## Concluding remarks

The rise of deep data encompassing all aspects of molecular and clinical phenotypes in increasingly larger human cohorts, allied with the rapid development of powerful computational analytical approaches, provides a platform to prioritise the directions of mechanistic studies. Original clinical questions ^123^ such as “why is there a near-universal involvement of the rectum?”, “why is mucosal inflammation different to CD?”, and “how does smoking protect?” and scientific ones such as “is there a specific antigenic trigger?”, “what is the relative importance of adaptive vs. innate immunity?”, and “what are the main mucosal factors that maintain the state of non-resolving inflammation in UC?” will emerge again and hopefully lead to better informed deductive (top-down logic) alongside the inductive (bottom-up logic) processes derived from these big datasets to fully understand the pathogenesis of UC.
